# Label-Free Myoglobin Biosensor Based on Pure and Copper-Doped Titanium Dioxide Nanomaterials

**DOI:** 10.3390/bios12121151

**Published:** 2022-12-08

**Authors:** Ahmad Umar, Mazharul Haque, Shafeeque G. Ansari, Hyung-Kee Seo, Ahmed A. Ibrahim, Mohsen A. M. Alhamami, Hassan Algadi, Zubaida A. Ansari

**Affiliations:** 1 Department of Chemistry, Faculty of Science and Arts, Najran University, Najran 11001, Saudi Arabia; 2 Promising Centre for Sensors and Electronic Devices (PCSED), Najran University, Najran 11001, Saudi Arabia; 3 Department of Materials Science and Engineering, The Ohio State University, Columbus, OH 43210, USA; 4 Centre for Interdisciplinary Research in Basic Sciences, Jamia Millia Islamia, New Delhi 110025, India; 5 School of Chemical Engineering, Jeonbuk National University, Jeonju 56212, Republic of Korea; 6 Department of Electrical Engineering, College of Engineering, Najran University, Najran 11001, Saudi Arabia

**Keywords:** Cu-doped TiO_2_, myoglobin, acute myocardial infarction, electrochemical biosensor

## Abstract

In this study, using pure and copper-doped titanium dioxide (Cu-TiO_2_) nanostructures as the base matrix, enzyme-less label free myoglobin detection to identify acute myocardial infarction was performed and presented. The Cu-TiO_2_ nanomaterials were prepared using facile sol–gel method. In order to comprehend the morphologies, compositions, structural, optical, and electrochemical characteristics, the pure and Cu-TiO_2_ nanomaterials were investigated by several techniques which clearly revealed good crystallinity and high purity. To fabricate the enzyme-less label free biosensor, thick films of synthesized nanomaterials were applied to the surface of a pre-fabricated gold screen-printed electrode (Au-SPE), which serves as a working electrode to construct the myoglobin (Mb) biosensors. The interference study of the fabricated biosensor was also carried out with human serum albumin (HSA) and cytochrome c (cyt-c). Interestingly, the Cu-doped TiO_2_ nanomaterial-based Mb biosensor displayed a higher sensitivity of 61.51 µAcm^−2^/nM and a lower detection limit of 14 pM with a response time of less than 10 ms.

## 1. Introduction

In the modern world, cardiovascular illnesses are the leading cause of death, with coronary artery disorders (CADs) accounting for about 32% of morbidity and mortality, with heart attacks and strokes accounting for 85% of cases [1,2,3,4,5,6,7,8]. Acute myocardial infarction (AMI), one of the cardiovascular illnesses, is a very severe condition brought on by a loss of blood flow for a variety of reasons [1,2,3,4,5,6,7,8,9,10]. AMI, sometimes referred to as a heart attack, happens when the major arteries leading to the heart become blocked, reducing or preventing blood flow to a portion of the myocardium for a number of reasons, including excessive cholesterol, obesity, high blood pressure, and diabetes [1,2,3,4,5,6,7,8,9,10,11,12,13,14,15]. Finally, heart cell necrosis results from the irreversible restriction of oxygen and nutrients in the blood supply. Cardiovascular diseases (CVD) cause more than 80% of deaths, and their prevalence is rising in both low- and high-income nations [3,4,5,6,7,8,9,10,11,12]. The world health organization (WHO) lists high levels of cardiac biomarkers in blood samples, unique chest discomfort, and a distinctive electrocardiogram (ECG) as indications of AMI [1,2,3,4,5,6,7,8,15,16,17,18,19,20,21,22,23,24,25,26,27,28,29,30]. The electrocardiogram (ECG), currently used to diagnose AMI, and the detection of characteristic clinical symptoms or cardiac biomarkers are critical for early clinical diagnosis and treatment which can save lives and reduce treatment costs [1,2,3,4,5,6,7,8]. Myoglobin (Mb), creatine kinase-MB (CK-MB), and cardiac troponins (cTnI and cTnT) are all often employed cardiac biomarkers [5,6,7,8,9,10,11,12,13,14,15,16,17,18,19,20,21,22].

Early detection of a specific cardiac biomarker in the blood with a high sensitivity is critical for optimal AMI treatment within 6 h after onset of symptoms [23,24,25,26,27,28,29,30,31,32]. Elevated myoglobin levels in the blood usually suggest that the patient has recently suffered a cardiac muscle injury. Although technological development has improved our understanding of the cardiovascular system, there are still certain limits in the diagnosis and treatment options available for CADs [30,31,32,33,34,35,36,37]. Nanotechnology has demonstrated tremendous potential for therapeutic use and has the ability to open up new avenues for the treatment and detection of cardiovascular diseases (CADs). In order to speed up patient treatment and reduce the death rate, early and accurate AMI detection methods needed to be developed. Nanotechnology helped with this, and with the development of effective, sensitive electrochemical biosensors based on customized nanomaterials, it is possible to detect cardiac biomarkers for AMI, such as myoglobin and troponins (I and T), thrombin, and so on [18,19,20,21,22,23,24,25,26,27,28,29,30,31,32,33,34,35,36,37]. Nanomaterials with modified surfaces and nanocomposites emerged as new viable tools for future therapeutics for CVDs. Various methods developed to fabricate nanomaterial-based AMI sensors utilizing intrinsic physiochemical properties; surface tailoring to enhance desirable cellular responses for cardiovascular detection show a significant potential and opportunities for clinical translation [38,39,40,41,42,43]. However, the research is still underway as translation of such sensors/materials for cardiovascular applications need a detailed understanding of both nanomaterials and biomedicine including the toxicity of nanomaterials [14,15,16,17,18,19,20,21,22,23,24,25,26,27,28,29,30,31,32,33,34,35,36,37].

Nanostructured transition metal oxide has drawn the research attention owing to its distinguishing characteristics such as low preparation cost, immense number of active sites, brief diffusion and distinctive reaction mechanism [38,39,40,41,42,43,44,45,46,47,48,49,50,51,52,53]. Among transition metal oxides, TiO_2_ is recognized as promising material because of better conductivity and its high degree of biocompatibility. TiO_2_ is an N-type semiconductor oxide with 3d^2^4s^2^ electronic configuration in the outermost shell where four valence electrons form covalent bond with O atom, resulting in high degree of chemically stability. Band gap of TiO_2_ is approximately 3.2 eV that can be tailored by inducing additional energy level to the conduction band via doping or imperfections to improve the sensing characteristics [38,39,40,41,42,43,44,45,46,47,48,49,50,51,52,53].

Recently, nanostructured TiO_2_ were used for a variety of biosensing applications such as to detect uric acid, cholesterol, glucose, troponin and myoglobin, as well as for cancer diagnosis [38,39,40,41,42,43,44,45,46,47,48,49,50,51,52,53]. Ag-doped TiO_2_ nanoparticles were used to detect amino acid tryptophan with glassy carbon electrode [39,40,41,42,43,44,45]. In addition, the TiO_2_ nanoparticles are used for enzyme-less detection of cholesterol which is considered as a critical indicator of clinical biochemistry [46].

In order to quickly measure biomolecules for illness diagnosis and environmental monitoring, electrochemical biosensors expand the possibility of real-time monitoring. Biosensors are now being investigated for use in the field of clinical and health care management [7,8,9,10]. The use of electrochemical sensors for process monitoring and control, particularly in situ process monitoring, is under increasing strain in a variety of disciplines, including biotechnology. Glassy carbon electrodes (GCE) are frequently used for biosensing applications and can be customized to meet specific needs; however, GCE is expensive, which prevents its usage in medical devices for diagnosis and healthcare that are economically viable [15,16,17,18,19,20,21,22,23,24,25,26,27,28,29,30]. Rapid diagnosis is made possible by the modification of GCE with nanostructured materials such as 2D graphene, semiconducting oxides, and composite nanomaterials, which also shows the potential for point-of-care (POC) devices with improved sensitivity and selective performance [30,31,32,33,34,35,36,37,38,39,40,41,42,43,44]. We developed Au-coated tri-electrode SPE on printed circuit board to lower the cost of the electrode since POC requires inexpensive, disposable electrodes/chips with three electrodes. Au helps with easy electron transfer characteristics and internal sensor features since it is an inert material and a good electrical conductor [30,32,49].

In this paper, we report the facile synthesis, detailed characterizations and fabrication of high-sensitive and selective enzyme-less label-free myoglobin biosensor based on Cu-TiO_2_ nanomaterials. To understand the selectivity of the fabricated biosensor, the interference study was also carried out with human serum albumin (HSA) and cytochrome c (cyt-c) as a feasibility study for the highly sensitive sensor for early CVD biomarker. The multianalyte sensing, fast detection, diagnostic, and point of care (POC) devices that are currently in high demand show tremendous potential with these electrochemical sensors.

## 2. Experimental Details

### 2.1. Materials

All of the chemicals, i.e., titanium isopropxide (TTIP), Isopropyl alcohol (IPA), copper acetate dihydrate (Cu(CH_3_COO)_2_. 2H_2_O), Myoglobin (100684-32-0), Cytochrome c (Cyt-c) (9007-436), Ammonium hydroxide (NH_4_OH, 28%), Nitric acid (HNO_3_), NaH_2_PO_4_.2H_2_O (98%) and Na_2_HPO_4_ (99%) were purchased from Sigma-Aldrich and utilized without any additional purification. Phosphate buffer solution (PBS) was prepared by mixing 0.1 M of each, i.e., NaH_2_PO_4_.2H_2_O (98%) and Na_2_HPO_4_ (99%) in 1:1 volumetric ratio. Deionised water (DI; 18 MΩ resistivity, Millipore) was used for all the solution preparations.

### 2.2. Synthesis of Pure and Cu-Doped TiO_2_ Nanomaterials

Pure and Cu-doped TiO_2_ nanomaterials were prepared as reported earlier [40]. In a typical reaction process, 1.69 M TTIP solution was prepared by dissolving 5 mL TTIP in 10 mL isopropyl alcohol. Three different concentrations (0.22 mM, 0.31 mM and 0.52 mM) of 200 mL Cu(CH_3_COO)_2_·2H_2_O solution, for each, were separately prepared in DI water under continuous stirring. After appropriate stirring, homogenous solutions of Cu(CH_3_COO)_2_·2H_2_O were obtained. In each of these solutions, pre-prepared 15 mL TTIP (1.69 M) was added drop-wise under continuous stirring; the total volume of 215 mL solution contains 80 mM of titanium precursor. Such concentrations provide three different solutions which ensure the Cu doping concentrations of 13 × 10^17^, 20 × 10^17^ and 32 × 10^17^ atoms/cm^3^, respectively, in the final solid product. During the reaction, the pH = 1.5 was set for the solution by drop-wise slow addition of nitric acid. The resultant solutions were stirred for 4 h at 80 °C and finally the obtained precipitates were decanted and washed with DI water and dried in an oven at 100 °C for 1 h. Pure TiO_2_ were synthesized with the same procedure except the Cu doping. The synthesized materials were further characterized by several techniques to investigate the structural, compositional, optical, morphological and sensing properties. The prepared samples were denoted as S0, S1, S2, and S3 for the pure TiO_2_, Cu-doped TiO_2_ (Cu =13 × 10^17^ atoms/cm^3^), Cu-doped TiO_2_ (Cu = 20 × 10^17^ atoms/cm^3^), and Cu-doped TiO_2_ (Cu =32 × 10^17^ atoms/cm^3^), respectively.

### 2.3. Characterizations of Pure and Cu-Doped TiO_2_ Nanomaterials

The synthesized materials were examined by several techniques. The structural properties and crystal characteristics were studied by x-ray diffraction (XRD; Ultima IV, Rigaku, X-ray diffractometer) using Cu-Kα (λ = 1.5406Å) X-ray source in the Bragg’s angle range of 20° to 80°. The chemical bonding, functional properties and chemical compositions of the prepared materials were studied by Fourier transform infrared (FTIR, Bruker’s Tensor 37 spectrophotometer) spectroscopy. The optical characteristics were obtained by acquiring UV absorption spectra in the range from 225 to 450 nm for the synthesized nanomaterials. Morphologies of the prepared materials were examined by Field emission scanning electron microscopy (FESEM, SU-70, Hitachi) at an accelerating voltage of 10 keV.

### 2.4. Fabrication and Characterizations of Myoglobin (Mb) Biosensors Using Pure and Cu-Doped TiO_2_ Nanomaterials

The myoglobin (Mb) biosensor devices were made on prefabricated 3 terminal gold-plated screen-printed electrodes (SPEs). The prepared nanomaterials were grinded well, and slurry of the obtained fine power (10 mg) was made by mixing the ethyl cellulose and butyl carbitol acetate (70:30 ratio). The prepared slurry was screen printed over the surface of the working electrode (4 mm diameter). The printed films on the SPEs were dried at 60°C for 4 h. By placing an optimal volume (30 µL) of analyte on the working electrode in SPE incorporating all three electrodes, i.e., the reference, counter, and working electrodes, three devices for each sample were fabricated and evaluated for different Mb concentrations [30,32,49].

The sensing properties of the fabricated biosensor were evaluated by cyclic voltammetry (CV; IVIUM’s Potentiostat) in the potential range from −1.0 V to +1.0 V at a scan rate of 100 mV/s. Solutions of different Mb concentrations were prepared in 0.1 M phosphate buffer solution (pH = 7.2). Scanning rates of 10–100 mV/s were used to evaluate charge transfer kinetics with a Mb concentration of 7 nM. To investigate the charge transfer characteristics, electrochemical impedance spectroscopy (EIS) study was performed simultaneously from 0.5 Hz–10^6^ Hz at 50 mV amplitude.

## 3. Results and Discussion

### 3.1. Characterizations and Properties of Pure and Cu-Doped TiO_2_ Nanomaterials

**Figure 1:**
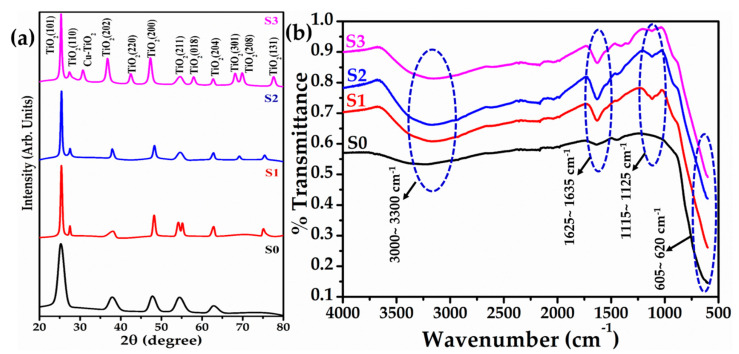
Typical (**a**) XRD pattern, and (**b**) FTIR spectra of pure and Cu-doped TiO_2_ nanomaterials.

Figure 1a depicts the XRD patterns of pure and Cu-doped TiO_2_ nanomaterials which exhibited various well-defined diffraction peaks. The peaks originated at 2θ = 25.40°, 27.33°, 37.71°, 42.41°, 47.38°, 54.60°, 57.86°, 62.66°, 68.06°, 69.79°, and 77.52° correspond to the TiO_2_(101), (110), (202), (220), (200), (211), (018), (04), (301), (208), and (131) planes, respectively. The appeared TiO_2_ peaks are well matched with the JCPDS card nos. 89-4921, 89-6975, 89-4203, and 89-4746. Interestingly, with higher Cu doping (S3), an extra XRD peak that originated at 2θ = 30.67° in sample S3 was observed, which can be related with the Cu-TiO_2_ composite.

The chemical bonding, functional properties and chemical compositions of the prepared pure and Cu-doped TiO_2_ nanomaterials were examined by Fourier transform infrared (FTIR) spectroscopy. Figure 1b depicts the typical FTIR spectra of pure and Cu-doped TiO_2_ nanomaterials which exhibited several well-defined peaks. For all the samples, the observed FTIR spectra exhibited a small absorption band in the range of 1625–1635 cm^−1^ along with a broad band in the range of 3000~3300 cm^−1^ which are assigned to the O–H bending and stretching modes of water, respectively [30,31,32,46]. The presence of a defined peak in the range of 1115–1125 cm^−1^, for all samples, can be correlated to the C-O stretching vibrations [32]. All the samples exhibited a broad peak in the range of 605~620 cm^−1^, a well-known finger print region for metal-oxygen (M-O; Ti-O)-related peak which clearly revealed that the synthesized materials are metal oxides [30,31,32,46]. 

**Figure 2:**
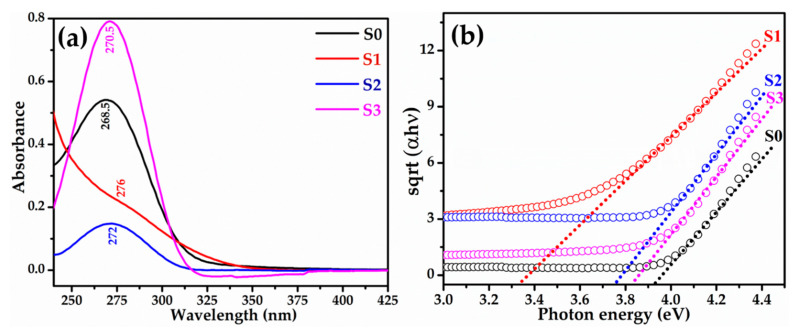
(**a**) Typical UV–vis spectra of pure and Cu-doped TiO_2_ nanomaterials and (**b**) their corresponding Tauc’s plots.

The optical properties of the synthesized pure and Cu-doped TiO_2_ nanomaterials were examined by UV-visible spectroscopy. Figure 2a depicts the typical UV–vis spectra of the prepared materials. The absorption peak for pristine TiO_2_ (S0) is observed at 268.5 nm, whereas Cu doping caused red shift of 2–8 nm with maximum for S1 sample. Figure 2b demonstrates the Tauc’s plot for the synthesized materials. The calculated bandgap values for the prepared materials are 3.92 eV, 3.33 eV, 3.76 eV, and 3.86 eV for S0, S1, S2, and S3, respectively. Interestingly, the sample S1 with doping concentration of 13 × 10^17^ atoms/cm^3^ exhibits lowest bandgap of 3.33 eV and provides maximum density of states.

**Figure 3:**
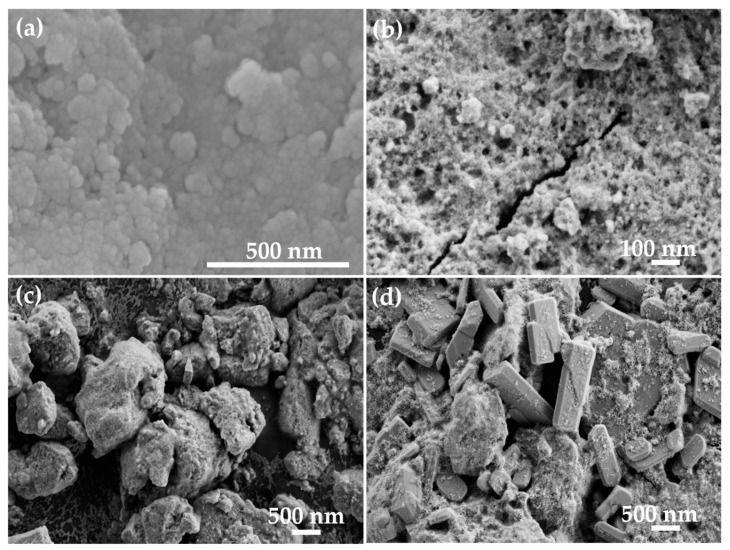
Typical FESEM images of pure and Cu-doped TiO_2_ nanomaterials (**a**) S0, (**b**) S1, (**c**) S2, and (**d**) S3 samples.

To study the morphological properties, the prepared pure and Cu-doped TiO_2_ nanomaterials were examined by field emission scanning electron microscopy (FESEM). Typical FESEM image of pure TiO_2_ is shown in Figure 3a, confirming the particle-shaped morphology of the synthesized material. Because the sizes of the particles are in the nanometer range, they are referred to as nanoparticles. The typical sizes of the nanoparticles are in the range of 30 ± 5 nm. With small doping of Cu ions into TiO_2_ (Cu =13 × 10^17^ atoms/cm^3^), the synthesized material does not change the morphology and possess the same particle-shaped structure in the nano-scale range (Figure 3b). The typical sizes of these nanoparticles are in the range of 25 ± 5 nm. With increasing the concentration of Cu doping into TiO_2_ (Cu = 20 × 10^17^ atoms/cm^3^), the nanoparticles agglomerate and form big particles as evident from the observed FESEM image (Figure 3c). Some small nanoparticles are also seen in the observed FESEM image. The typical sizes of the observed particles are in the range of 800 ± 100 nm. At higher Cu ions doping in the TiO_2_ (Cu =32 × 10^17^ atoms/cm^3^), micro-sized large rectangular brick-like morphologies formed (Figure 3d). The typical sizes of the brick-like morphologies are in the range of 1–1.5 µm.

### 3.2. Fabrication and Characterizations of Mb Biosensor Based on Pure and Cu-Doped TiO_2_ Nanomaterials

**Figure 4:**
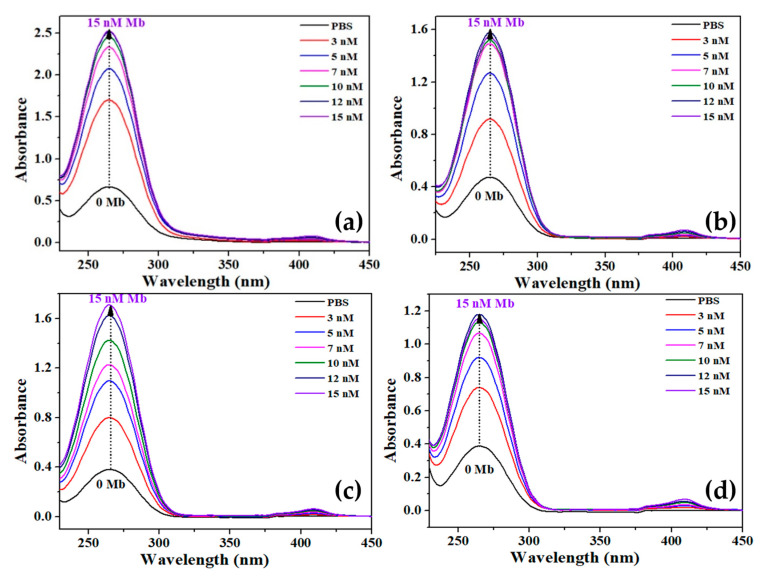
UV–vis absorption spectra of pure and Cu-doped TiO_2_ nanomaterials with various concentrations of Mb, (**a**) S0, (**b**) S1, (**c**) S2, and (**d**) S3.

Figure 4 exhibits the typical UV–vis absorption spectra of pure and Cu-doped TiO_2_ nanomaterials with various concentrations (3 nM to 15 nM) of Mb. The absorption spectra were recording in PBS solution with pH = 7.4 in presence of 10 μg/mL pure and Cu-doped TiO_2_ nanomaterials. For all the samples, dominant absorption peaks appeared in the range of 268–276 nm, while small and suppressed peaks were seen in the range of 412–415 nm. The absorption peaks originated in the range of 268–276 nm due to the presence of Mb protein, while the peaks in the region of 412–415 nm refer to the Soret band because of the presence of heme. In the presence of Mb, the presence of tiny peak in the range of 412–415 nm suggests an increase in oxidation and interfacial resistance. Interestingly, it was observed that with increasing the concentration of Mb in the solution, the absorption intensity increases. Further, the full width at half maxima widens with Mb concentration suggesting the increase in electron cloud owing to the oxidation of Mb. In addition, the increase in intensity of Soret band with increasing the Mb concentrations in the solution suggest the enhancement of chromophores, i.e., free Mb molecules that do not form conjugation with nanomaterials, which clearly revealed that the native state of protein remains intact.

**Figure 5:**
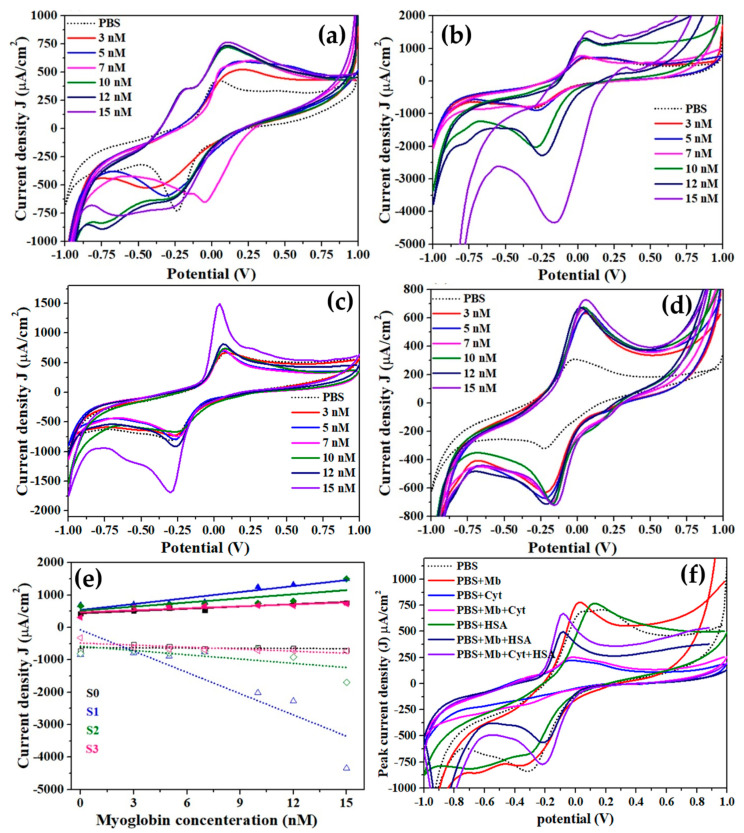
CV curves obtained at 100 mV/s for pure (**a**) and (**b**–**d**) Cu-doped TiO_2_ nanomaterials in absence and presence of various concentrations of Mb. I Graph of oxidation peak current vs. Mb concentration (experimental data represented by symbols and best fit in dotted line), and (**f**) Interference study with cyt-c and human serum albumin.

Through the use of cyclic voltammetry, the sensing capabilities of the fabricated Mb biosensor were investigated for a range of Mb concentrations (from 3 nM to 15 nM). Figure 5 a–d depicts the typical CV responses of the fabricated biosensor using pure and Cu-doped TiO_2_ nanomaterials in absence and presence of various concentrations of Mb. Interestingly, with increasing the concentrations of Mb, the oxidation and reduction peak currents are increases. Thus, for all fabricated electrodes based on pure (S0) and Cu-doped TiO_2_ (S1–S3) nanomaterials, the values of both reduction and oxidation peak current increases directly with Mb concentration. At 3 nM Mb concentration, the observed oxidation/reduction peak potentials for pure TiO_2_ (S0) nanoparticles are 0.18 V and −0.42 V, respectively (Figure 5a). A shoulder in the CV peak of S0 sample for both the oxidation and reduction peaks on the low energy side suggests that the material has two phases that interact with Mb. With increased Mb concentration, the redox peak potential decreases. The oxidation peak for S1 samples was 0.04 V, while the reduction peak was −0.3 V. As illustrated in Figure 5b, the redox peak potential shifts noticeably with Mb concentration at high values. As shown in Figure 5c,d, the oxidation peak currents for S2 and S3 samples were 0.06 V and −0.22 V, respectively, with a small shift towards low values. The change in internal resistance caused by rising Mb concentration is linked with shifts in potentials [33]. The sensitivity was estimated as the slope of the calibration curves of oxidation peak current density (J) versus Mb concentration (Figure 5e), initially increased with Cu doping compared to that of pure TiO_2_ and then reduced at high doping concentration (Table 1).

The fact that the difference in redox peak potential for all samples is always less than 0.59 V indicates that the reaction is reversible. As a result, the sensors can be utilized multiple times, thus lowering the cost of diagnosis. Based on the observed results, the calculated sensitivities for the fabricated biosensors obtained with pure TiO_2_ and Cu-doped TiO_2_ nanomaterials with various doping concentrations were 23.43 µAcm^−2^/nM, 61.51 µAcm^−2^/nM, 42.45 µAcm^−2^/nM, and 20.43 µAcm^−2^/nM for the samples S0, S1, S2, and S3, respectively. In addition, the observed limits of detection (LODs) for the fabricated biosensors were 153 pM, 14 pM, 32 pM, and 68 pM for the samples S0, S1, S2, and S3, respectively. Among all the fabricated biosensors, sample S1-based biosensor exhibited the highest sensitivity (61.51 µAcm^−2^/nM) and lowest detection limit (14 pM) while S3 showed the lowest sensitivity. The highest sensitivity of the S1 sample might be due to the high surface to volume ratio of the applied material on the working electrode, acting as a potential scaffold and providing larger sites for the Mb interaction which contributed to the high-sensitivity. The sensing characteristics of the fabricated Mb biosensors based on pure and Cu-doped TiO_2_ nanomaterials were compared with the reported Mb biosensors, which clearly confirmed that our fabricated biosensor based on S1 sample exhibited the best sensing performance (Table 2).

One of the reasons for the highest sensitivity of S1 may be the smallest crystal size of grown nanoparticles (Figure 3b), which has the highest surface area to volume ratio and offers a high density of states for the interaction, whereas further increasing the Cu doping concentration results in the formation of larger crystals with lower sensitivity (Figure 3c,d). According to FESEM (Figure 3b), Cu doping of 13 × 10^17^ atoms/cm^3^ in TiO_2_ matrix shows the growth of relatively small size, which is caused by strain in the crystal as a result of 13.37% lattice mismatch. This offers a high density of states for the adsorption, a big surface to volume ratio, the maximum sensitivity, and the lowest LOD. However, as the concentration of Cu in the TiO_2_ matrix rises, larger crystals (about five times larger in size) form, which have a poor surface area to volume ratio and so exhibit relatively low sensitivity.

To examine the specificity and selectivity, interference studies for the fabricated Mb biosensors were examined in presence of cytochrome c, a hemeprotein, and a human serum albumin protein, which is abundant in the blood serum. Figure 5f exhibits the interference studies of the fabricated sensors. Interference experiments using cytochrome c and human serum albumin (HSA) protein show unique redox peak currents and potentials, as shown in Figure 5f for each analyte separately and a 1:1 volumetric mixture of 7 nM Mb and 7 nM cyt-c. PBS, Mb, and cyt-c had oxidation peak potentials of 0.04 V, 0.02 V, and −0.02 V, respectively, whereas reduction peak potentials were −0.3 V, −0.28 V, and 0.16 V. The oxidation peak potential of −0.04 V in 1:1 mixed solution is fairly modest; however, the lowered potential of −0.32 V shows a specific response of the sensor to Mb in the presence of cyt-c. The human serum albIn (HSA) (purple curve) has a different oxidation and reduction peak at −0.092 V and a different reduction peak at −0.216 V compared to Mb. Interestingly, it was observed that the Mb has a 1.56 times higher oxidation current than cyt-c and a 1.13 times higher oxidation currI than HSA, confirming the high selectivity and sensitivity of the fabricated Mb biosensor.

**Figure 6:**
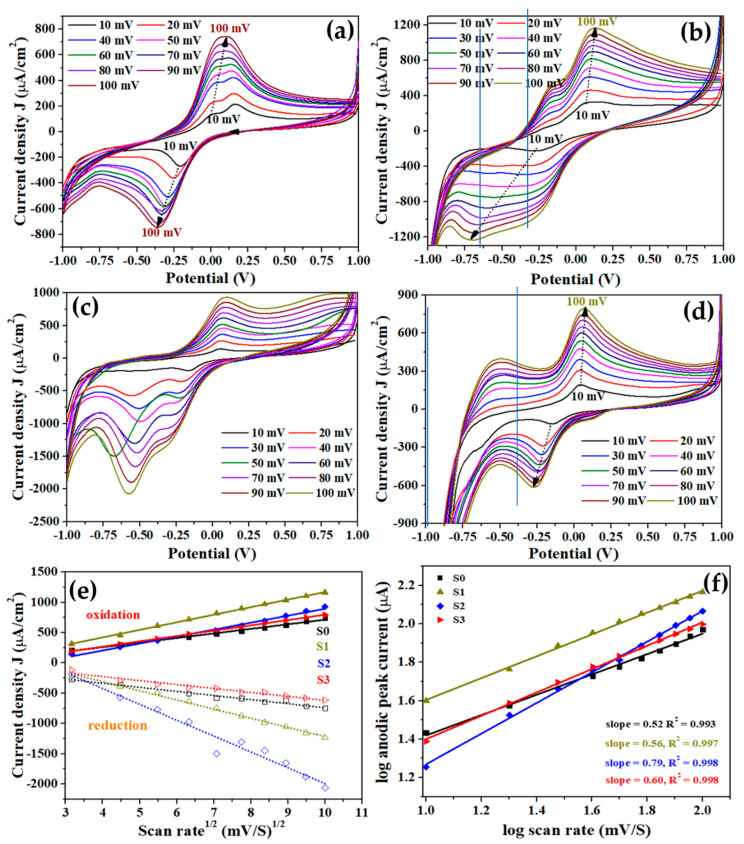
CV curves for pure (**a**) and (**b**–**d**) Cu-doped TiO_2_ nanomaterials at various scan rates (10 to 100 mV/s) in the presence of 7nM Mb; (**e**) plot of redox peak current against square root of scan rate, and (**f**) plot of log of oxidation peak current against log of scan rate. Experimental data points are represented by symbols and dotted line indicates best fit.

Figure 6 exhibits the typical CV curves of the modified electrodes with pure and Cu-doped TiO_2_ nanomaterials at various scan rates (10 to 100 mV/s) in the presence of 7 nM Mb. The CVs at various scan rates of the modified electrodes were used to analyze the charge transfer kinetics of the constructed devices. Interestingly, it was observed from the CV analysis of all the fabricated electrodes based on pure and Cu-doped TiO_2_ nanomaterials that with increasing the scan rates, the redox peaks also enhanced. For the S0 sample, the CV graphs indicate that there was a split in the oxidation peaks at lower scan rates (up to 50 mV/s) which merged to form a single peak at high scan rates (Figure 6a). The CV analysis of the S1 sample apparently revealed a clear shoulder towards the low energy side which was accompanied with a broad reduction peak with a width of 0.44 V and a primary oxidation peak separated by 0.13 V (Figure 6b). For S2 sample, a shoulder emerged in the reduction peak (Figure 6c); however, S3 sample possessed two distinct oxidation peaks that appeared at −0.50 V and 0.11 V (Figure 6d).

With increased scan rate, there is a little shift in redox peak potential. The oxidation peak shifted slightly toward high energy, but the reduction peaks red shifted, i.e., towards negative potential. At varying scan rates, the shift in peak positions indicates a change in reaction rate and kinetics [43]. At a scan rate of 100 mV/s, an increase in current directly proportional to scan rate supports Faradic interfacial redox processes with substantially faster electron and ion movement [44].

In general, splitting of oxidation peaks is observed for samples S0, S1, and S3 (Figure 6a,b and d) which implies the presence of two different phases of TiO_2_, probably TiO_2_(101) and TiO_2_ (200), that are contributing during the reaction. In S0 (Figure 6a), the splitting implies two phases contributing at low scan rates, but is not differentiable at high scan rates. The oxidation peak in S1–S3 is widened at a low scan rate of 10 mV/s and is clearly divided into two peaks. The existence of a high concentration of Cu atoms in the matrix, which at high Cu concentration results in the formation of Cu-TiO_2_ mixed phase associated with variable oxidation potential, can be correlated with S3 at high scan rates for S2 (Figure 1a).

Figure 6e shows a linear relationship between peak current and square root of scan rate, indicating a diffusion-controlled process. In addition, the slope and correlation coefficient of the graph of log-scan rate vs. log-peak current obtained from Figure 6f are approximately 0.52–0.79 and 0.99, respectively, indicating that the reaction is diffusion-controlled. The Randles–Sevcik equation, shown below (equation 1), was used to calculate the diffusion coefficient for all samples, and the results are shown in Table 2.

i_p_ = 0.4463*A*C*F*n^3/2^·(F·νD/RT)^1/2^ (1)

**Figure 7:**
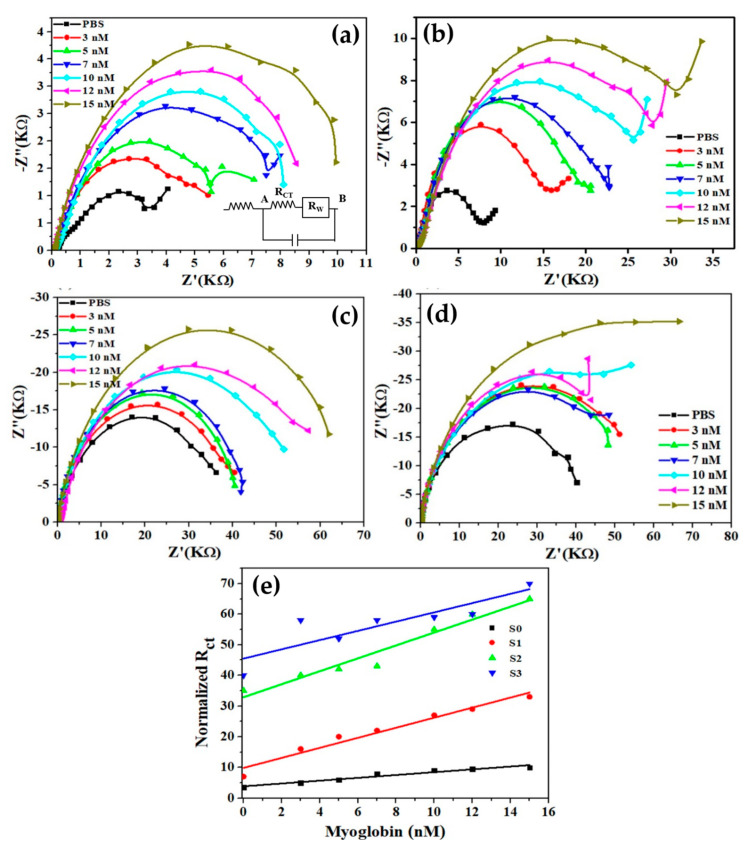
Series of Nyquist plots obtained from the pure (**a**) and (**b**–**d**) Cu-doped TiO_2_ nanomaterials in absence and presence of various concentIions of Mb. (**e**) R*ct* values for different Mb concentration.

The electrochemical impedance studies for all samples (S0–S3) were carried out by standardizing real and imaginary values of impedance using the Nyquist plot displayed in Figure 7 to explore the influence of Mb on charge transfer resistance (R*ct*). The value of charge transfer resistance, i.e., R*ct*, is determined by the diameter of the semicircle, which increases with increasing the Mb concentration in all samples (S0–S3) (Figure 7a–d). The increase in R*ct* values in the presence of Mb was expected since it forms a conjugate with nanomaterials during the sensing process. Figure 7e exhibits the curves for normalized R*ct* values vs. Mb concentrations. As observed in Figure 7e, sample S1 exhibited higher sensitivity compared to the sensor fabricated based on pure TiO_2_ (S0). However, other samples, S2 and S3, possess higher R*ct* but lower sensitivity. The result findings indicate that the proposed sensor system is very much capable of detecting Mb in PBS. R*ct* values increased as Mb concentration increased due to a substantial amount of Mb adsorption inhibiting the interfacial charge transfer mechanism.

The equivalent circuit of Nyquist curves is shown in the inset of Figure 7a. Here, point A represents the surface of the film, and point B corresponds to the solid liquid interface that constitutes the outermost layer of the double layer. Rw stands for diffusion impedance, or Warburg impedance, which is related to species diffusion and has a high value at low frequencies but a negligibly low value at high frequencies. Warburg impedance, on the other hand, is dependent on reaction kinetics and diffusion. The charge transfer is driven by the potential at point B, which results in a change in potential at different Mb concentrations in the solution.

**Figure 8:**
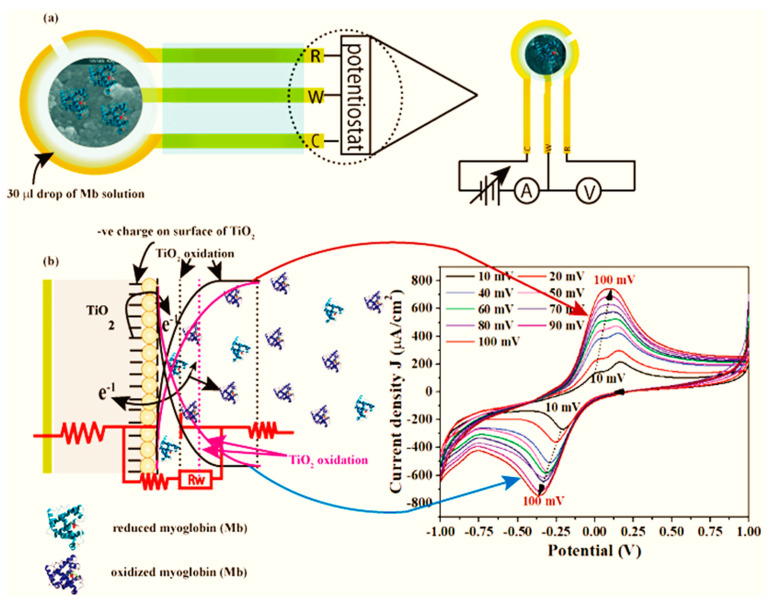
(**a**) Fabrication of Mb biosensor based on pure and Cu-doped TiO_2_ nanomaterials, and (**b**) Mb sensing mechanism.

A proposed sensing mechanism for the fabricated Mb biosensor based on the obtained results is depicted in Figure 8. Figure 8a shows the typical fabrication process in which the functional nanomaterials, i.e., pure and Cu-doped TiO_2_, were coated onto the SPE electrode containing all three electrodes (working, counter and reference) in a single device. The functional nanomaterial modified electrode was used as a working electrode. For the sensing, 30 µL of Mb solution was immobilized onto the surface of the working electrode. Figure 8b exhibits a likely electron transfer pathway during sensing of Mb. As the myoglobin (Mb) is an iron containing heme-protein, during detection process, the Fe^3+^/ Fe^2+^ redox reactions occurred on the electrode surface. During reaction process, once the electrode potential crosses the E_1/2_ value, an electron is transported from TiO_2_ to Mb, reducing Fe^3+^ to Fe^2+^, causing the oxidation current to grow. This is reflected in a smaller depletion region and easier ion transport from the electrode to the bulk solution. On the other hand, when the applied voltage achieves equilibrium during a negative sweep, electron transfer from Mb to the electrode surface occurs, oxidizing Fe^+2^ to Fe^+3^ and releasing one electron to TiO_2_, increasing the amplitude of the reduced current. Therefore, ions diffuse from the bulk to the electrode surface, causing a drop in current. This is a single electron transfer mechanism that is reversible.

## 4. Conclusions

A highly sensitive enzyme-less Mb biosensor was successfully fabricated using pure and Cu-doped TiO_2_ nanomaterials. The nanomaterials were synthesized via simple sol–gel process and characterized by several techniques in order to investigate the structural, composition, optical, morphological and sensing properties. The structural properties observed by XRD clearly revealed the good crystallinity for the synthesized materials. The synthesized pure and Cu-doped TiO_2_ nanomaterials were employed as a functional material to modify the working electrodes to fabricate the Mb biosensor over a wide range of Mb concentrations, i.e., from 3 nM to 15 nM. The calculated sensitivities for the fabricated biosensors based on pure TiO_2_ and Cu-doped TiO_2_ nanomaterials, with various doping concentrations, were 23.43 µAcm^−2^/nM, 61.51 µAcm^−2^/nM, 42.45 µAcm^−2^/nM, and 20.43 µAcm^−2^/nM for the samples S0, S1, S2, and S3, respectively. In addition, the observed limits of detection (LODs) for the fabricated biosensors were 153 pM, 14 pM, 32 pM, and 68 pM for the samples S0, S1, S2, and S3, respectively. Based on the observed results, it can be incurred that the Cu-TiO_2_ nanomaterials are promising candidates as a potential portable sensing platform for the future point-of-care diagnostics.

## Figures and Tables

**Table 1:** Estimated sensing parameters for the fabricated Mb biosensor based on pure and Cu-doped TiO_2_ nanomaterials.

Samples	Peak Oxidation Potential (V)	Peak Reduction Potential (V)	Diffusion Coefficient (D) (×10^−9^ cm^2^/s)	Sensitivity (µAcm^−2^/nM)	LOD Value (pM)
**S0**	0.04–0.08	−0.24, −0.28	0.76	23.43	153
**S1**	0.04–0.06	−0.32–(−0.16)	1.87	61.51	14
**S2**	0.08–0.04	−0.30	1.19	42.45	32
**S3**	0.06	−0.22	0.87	20.43	68

**Table 2:** Comparison of the sensing performance of fabricated Mb sensors with other reported Mb sensors fabricated based on various sensing matrixes.

Sensing Matrix	Method	Linear range	LOD	Ref.
Anti-MYO/4-ATP SAM/Au	EIS	350 ngmL^−1^–17.5 μgmL^−1^	5.5 ngmL^−1^	[27]
Ab-MYO/AuNps/APTES/ITO	EIS	10 ngmL^−1^–1μgmL^−1^	2.7 ngmL^−1^	[28]
MBA/AuNps/RGD/GRCOOH/GCE	EIS	0.0001–0.2 gL^−1^	26.3 ngmL^−1^	[29]
TiO_2_-Mn NPs/SPE	CV and EIS	3 nM–15 nM	0.013 nM	[30]
Fiber-optics	SPR	15–30 ngml^−1^	2.9 ngmL^−1^	[31]
ZnO-Mn NPs/SPE	CV and EIS	0.024–2.4 ngmL^−1^	0.35 nM	[32]
MIP/MWCNT/GCE	DPV	1 μgmL^−1^–0.1 mgmL^−1^	0.17 μgmL^−1^	[33]
DApt/Exo I/Au	DPV	0–1.4 μgmL^−1^	0.47 ngmL^−1^	[34]
AuNp@rGO/SPE	DPV	1 ngmL^−1^–1400 ngmL^−1^	0.67 ngmL^−1^	[35]
ZnO-Cu NPs/SPE	CV and EIS	3 nM–15 nM	0.46 nM	[49]
Cu-TiO_2_ NPs/SPE	CV and EIS	3 nM–15 nM	S0:0.153 nM S1:0.014 nM S2: 0.032 nM S3:0.068 nM	This work

## Data Availability

Not applicable.
